# Inhibition of Cytochrome P450 Activities by Extracts of *Hyptis verticillata* Jacq.: Assessment for Potential HERB-Drug Interactions

**DOI:** 10.3390/molecules23020430

**Published:** 2018-02-15

**Authors:** David Picking, Bentley Chambers, James Barker, Iltaf Shah, Roy Porter, Declan P Naughton, Rupika Delgoda

**Affiliations:** 1 Natural Products Institute, University of the West Indies, Mona, Kingston 7, Jamaica; Davidpicking63@gmail.com; 2 Tropical Medicine Research Institute, University of the West Indies, Mona, Kingston 7, Jamaica; bentley.chambers@uwimona.edu.jm; 3 School of Life Sciences, Pharmacy & Chemistry, Kingston University, Kingston upon Thames, UK; J.Barker@kingston.ac.uk (J.B.); D.Naughton@kingston.ac.uk (D.P.K.); 4 College of Science, United Arab Emirates University, Al-Ain 009713, United Arab Emirates; altafshah@uaeu.ac.ae; 5 Department of Chemistry, University of the West Indies, Mona, Kingston 7, Jamaica; roy.porter@uwimona.edu.jm

**Keywords:** cytochrome P450, *Hyptis verticillata* Jacq., enzyme inhibition, herb-drug interaction, drug-herb interaction, adverse drug reaction, ADR, chemoprevention, antioxidant

## Abstract

Understanding the potential for adverse drug reactions (ADRs), from herb-drug interactions, is a key aspect of medicinal plant safety, with particular relevance for public health in countries where medicinal plant use is highly prevalent. We undertook an in-depth assessment of extracts of *Hyptis verticillata* Jacq., via its impact on activities of key cytochrome P450 (CYP) enzymes (CYPs 1A1, 1A2, 1B1, 3A4 and 2D6), its antioxidant properties (determined by DPPH assays) and chemical characterisation (using LC-MS). The dried plant aqueous extract demonstrated potent inhibition of the activities of CYPs 1A1 (7.6 µg/mL), 1A2 (1.9 µg/mL), 1B1 (9.4 µg/mL) and 3A4 (6.8 µg/mL). Further analysis of other crude extracts demonstrated potent inhibition of CYP1A2 activity for a dried plant ethanol extract (1.5 µg/mL), fresh plant ethanol extract (3.9 µg/mL), and moderate activity for a fresh plant aqueous extract (27.8 µg/mL). All four extracts demonstrated strong antioxidant activity, compared to the positive control (ascorbic acid, 1.3 µg/mL), with the dried plant ethanol extract being the most potent (1.6 µg/mL). Analysis of the dried plant aqueous extract confirmed the identity of seven phytochemicals, five lignans and two triterpenes. Individual screening of these phytochemicals against the activity of CYP1A2 identified yatein as a moderate inhibitor (71.9 μM), likely to contribute to the plant extract’s potent bioactivity. Further analysis on the impact of this plant on key drug metabolizing enzymes in vivo appears warranted for likely ADRs, as well as furthering development as a potential chemopreventive agent.

## 1. Introduction

The use of medicinal plants continues to be high, with the World Health Organisation (WHO) estimating 80% prevalence in developing countries [1], and many people continuing to rely on them as their main or only type of primary healthcare [2]. We previously reported that 73% of Jamaican adults use medicinal plants to treat illness and maintain health [3]. From the same research, we also reported that 27% of Jamaican medicinal plant users take pharmaceutical drugs concomitantly. A survey of cancer patients attending the oncology and urology clinics at the University Hospital of the West Indies in Kingston, and two earlier surveys of Jamaican prescription drug users identified high levels of herb-drug use (80% [4] 80% [5] and 81% [6], respectively), while awareness of such herb-drug use amongst health care professionals was low across all four surveys (13% [5], 15% [4], 18% [6] and 19% [3], respectively). High levels of concomitant herb-drug use and low physician awareness are reported elsewhere. For example, a survey of 381 patients in Norwegian general practice reported 44% prevalence for medicinal plant use, 45% concomitant use with pharmaceutical drugs, and 25% awareness amongst attending physicians [7].

Adverse drug reactions (ADRs) resulting from the altered metabolism of one drug caused by the interaction of a second drug are well researched and documented [8]. Herb-drug interactions, however, are far less researched, particularly in developing countries, where medicinal plant use is most prevalent [1,3]. Understanding the clinical impact and potential risk of ADRs, resulting from herb-drug interactions, is a critical public health issue, and a key aspect of medicinal plant safety. However, most of the plant-based research, to date, has been completed in developed countries, predominantly using *in vitro* studies, but, also with increasing numbers of clinical studies. Clinical studies of some of the most commonly researched plants include garlic (*Allium sativum*), St. John’s wort (*Hypericum perforatum*), *Panax ginseng*, and *Ginkgo biloba* [9].

Our laboratory has been working to address both the imbalance in research output from the Caribbean, and the potential public health risk, by testing medicinal plants, commonly used in Jamaica, for their impact on key drug metabolizing enzymes [10,11,12]. Plants tested include *Artocarpus altilis* [13], *Peperomia pellucida* [14], *Petiveria alliacea* [15] and *Picrasma excelsa* [16].

This latest study set out to investigate the inhibitory impact of various extracts of *H. verticillata* on a number of key drug metabolizing CYP450 enzymes, its characterisation and further impact on CYP1A2, representing the first such *in vitro* evaluation for this plant, and to add to the growing list of screened Caribbean medicinal plants. CYP1 inhibitions and associated antioxidant activities have also been linked with chemopreventive potential and thus we also carried out an in vitro antioxidant screen [8]. *H. verticillata* was previously identified as one of the top 25 medicinal plants used by Jamaicans in a TRAMIL survey [3,17]. TRAMIL, a Caribbean-wide applied research programme, scientifically evaluates and documents the efficacy and safety of medicinal plant remedies used for primary healthcare [18]. An earlier comprehensive review of this plant details its extensive traditional uses, phytochemistry pharmacology and toxicology [19]. For example, traditional uses documented in Jamaica include, bronchitis, cold, colic, fever, indigestion, itchy skin, mucous congestion, arthritis, tonsillitis, and fibroids [19]. In our earlier survey, documenting concomitant use, respondents identified their use of *H. verticillata* in combination with a number of pharmaceutical drugs, including aspirin, hydralazine, hydrochlorothiazide, and metformin. Respondents identified using *H. verticillata* for different health conditions to those for which they were prescribed a pharmaceutical drug(s) [3].

## 2. Results

### 2.1. Inhibition of CYP Activity by Aqueous Extract of Dried Aerial Plant Material 

**Figure 1:**
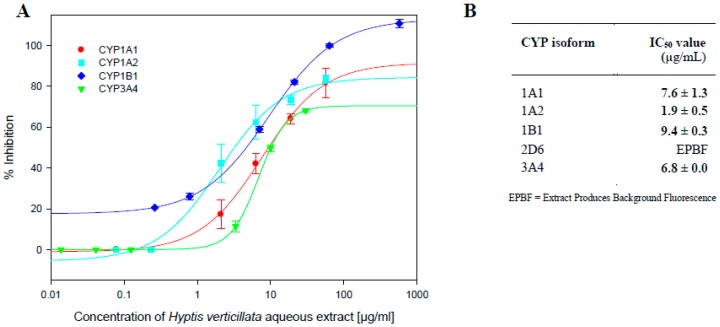
(**A**) Impact of *H. verticillata* aqueous extract on CYP activities. Human CYPs 1A1, 1A2 catalyzed 7-ethoxy-3-cyanocoumarin (CEC) (0.5, 5.0 μM respectively) metabolism, CYP1B1 catalyzed 7-ethoxyresorufin (ERes) (0.37 μM) metabolism and CYP3A4 catalyzed 7-benzyloxy-4-trifluoromethylcoumarin (BFC) (50 μM) metabolism were determined in the presence of varying concentrations of reconstituted freeze-dried aqueous extract of *H. verticillata* ranging from 0.02 to 575 µg/mL, as described in the Materials and Methods section. Control enzyme activity (mean ± SEM) for CYPs 1A1, 1A2, 1B1 and 3A4 was 1.43 ± 0.2, 1.49 ± 0.001, 0.95 ± 0.001 and 0.86 ± 0.000 (µM/min/pmol) of CYP, respectively. Data are expressed as mean percentage of enzyme activity for experiments carried out in triplicate; (**B**) Summary of results.

The impact of an aqueous extract of the dried aerial plant on the activities of a number of CYP enzymes was investigated, and a summary of the results shown in Figure 1A,B. Best-fit non-linear plots were fitted to the data points using the 4-parameter logistic (4PL) non-linear regression model (SigmaPlot) and IC_50_ values determined for four of the five enzymes tested. The aqueous extract of *H. verticillata* potently inhibited the activity of all four enzymes, with the concentration needed to inhibit 50% of activity, IC_50_ values all being under 10 µg/mL: CYPs 1A2 (1.9 µg/mL), 1A1 (7.6 µg/mL), 1B1 (9.4 µg/mL) and 3A4 (6.8 µg/mL). It was not possible to measure the aqueous extract’s impact on CYP2D6 due to a high level of background fluorescence produced by the extract (Figure 1A,B).

### 2.2. Inhibition of CYP1A2 Activity by Different Crude Extracts of H. verticillata (Aerial Plant)

Best-fit non-linear plots were fitted to the data points using the 4-parameter logistic (4PL) non-linear regression model (SigmaPlot) and IC_50_ values determined for four aerial plant extracts, aqueous dried and fresh and ethanol, dried and fresh. Both fresh and dry ethanol extracts and the dry aqueous extract demonstrated potent inhibition of CYP1A2 activity with the dry plant ethanol extract demonstrating the most potent inhibition (1.5 µg/mL). The dry aqueous extract potently inhibited CYP1A2 activity and was close in potency at 1.9 µg/mL to the dry plant ethanol extract and was more potent than the fresh plant ethanol extract (3.9 µg/mL). The fresh plant aqueous extract moderately inhibited CYP1A2 activity (27.8 µg/mL).

### 2.3. Antioxidant Assays

**Figure 2:**
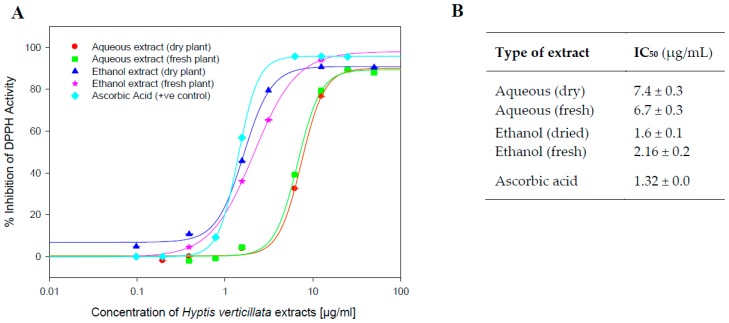
(**A**) DPPH antioxidant assay for *H. verticillata* extracts. Antioxidant capacity was determined in the presence of varying concentrations of reconstituted freeze-dried aqueous extracts and ethanol extracts of *H. verticillata* from both fresh and dry aerial plant material ranging from 0.097 to 50 µg/mL, as described in the Materials and Methods section. Positive control activity was determined in the presence of varying concentrations of known antioxidant, ascorbic acid, ranging from 0.097 to 50 µg/mL. Data are expressed as Mean Percentage Antioxidant Activity (% Inhibition of DPPH) for duplicate assays for at least two independent experiments. (**B**) Summary of results.

The 1,1-diphenyl-2-picrylhydrazyl (DPPH) assay was used to measure the antioxidant capacity of four different extracts of the aerial parts of *H. verticillata* (Figure 2). The reduction of DPPH to hydrazine produces a colour change from purple to yellow, which is measured spectrophotometrically and compared with the known antioxidant, ascorbic acid, as described in the Materials and Methods section.

All four extracts demonstrated strong antioxidant activity in comparison to the positive control, with the ethanol dried plant extract showing the most potent activity, almost directly comparable to that of ascorbic acid (Figure 2).

### 2.4. Standardization of Dried Aerial Plant Aqueous Extract by RP-HPLC and LC-MS 

#### 2.4.1. RP-HPLC

**Figure A1:**
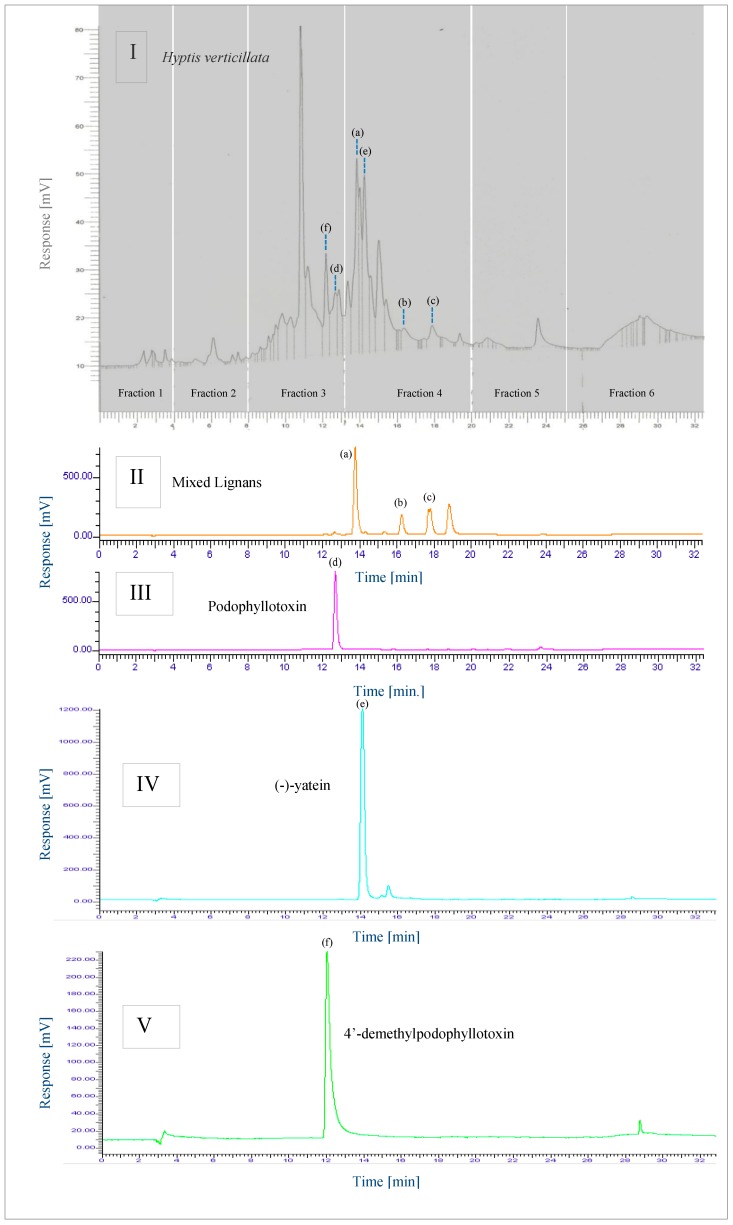
RP-HPLC analysis of reconstituted aqueous extract of *H. verticillata* (**I**) with comparison to standards: 5 lignan mix (**II**) [6 mg/mL]; podophyllotoxin (**III**) [7.8 mM]; (-)-yatein [10 mM] (**IV**) and 4′-demethylpodophyllotoxin [2 mM] (**V**). Separations were achieved using a Perkin-Elmer Series 200 chromatograph, Waltham, MA, USA fitted with a Chromsep C18, 250 × 4.6 mm, 5 µm column and C18 guard column. An injection volume of 20 uL was independently run for *H. verticillata* (**I**) and each of the standards (**II**, **III**, **IV** and **V**) for 34 min at a flow rate of 1 mL/min. at wavelength 290 nm at an ambient temperature of 18 °C. The mobile phase consisted of methanol and water using gradient elution: 20% methanol for 2 min increased to 60% linearly over 4 min and held for 2 min. Methanol was further increased to 80% linearly over 9 min and held for 7 min. The column was washed by running 100% methanol for 8 min then decreased to 20% to equilibrate the column for the next injection.

An initial separation of the aqueous extract of *H. verticillata* (dried aerial material) was undertaken using RP-HPLC, through which fractions were collected using a Gilson FC 203B fraction collector and concentrated on a Savant SpeedVac concentrator (Appendix A, Figure A1), and subsequent analysis undertaken using LC-MS (Appendix B, Table A1), as described in the Materials and Methods section.

#### 2.4.2. LC-MS

The exact mass to charge ratio (*m*/*z*) values of all ion species detected by a high resolution LC-MS were recorded, interpreted, and compared with the molecular weights of all known metabolites that have been identified to date for the aerial parts of *H. verticillata* [19,20,21] and fifteen potentially positive spectral results were identified (Table 1) (Appendix B, Table A1). In each case, mass differences between calculated and measured were within calibrant tolerance. The use of exact mass and adduct measurement confirms in most cases the identity of the compounds.

**Figure 3:**
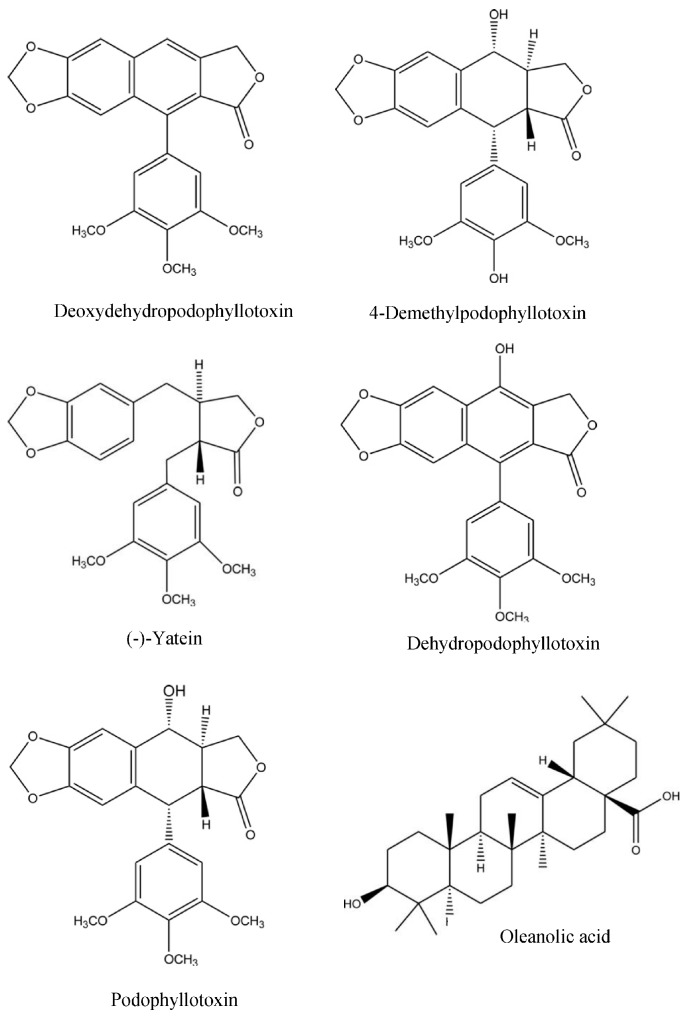
Structures of phytochemicals identified in the aqueous extract of *H. verticillata* (aerial, dried).

**Figure A2:**
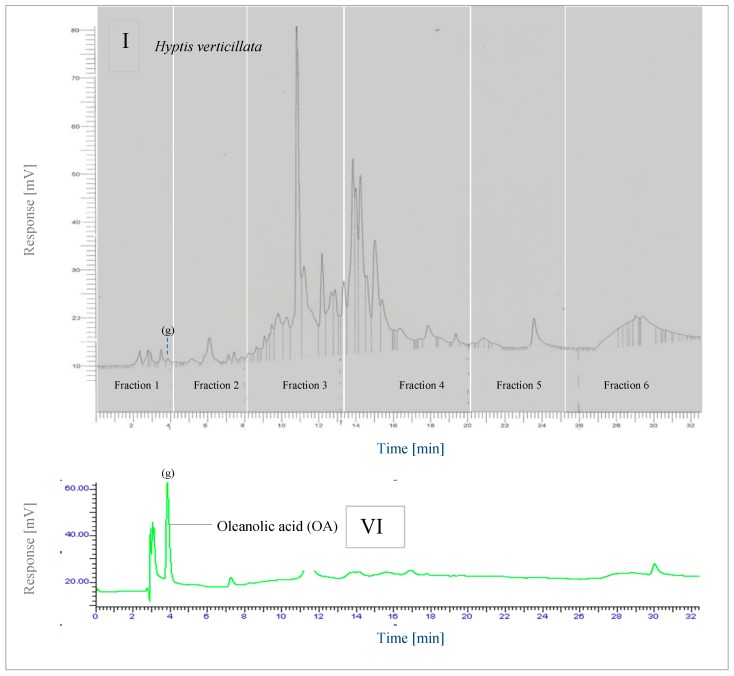
RP-HPLC analysis of reconstituted aqueous extract of *H. verticillata* (**I**) with comparison to standard: oleanolic acid (**VI**) [7.8 mM]. Separations were achieved using a Perkin-Elmer Series 200 chromatograph, Waltham, MA (USA) fitted with a Chromsep C18, 250 × 4.6 mm, 5 µm column and C18 guard column. An injection volume of 20 uL was independently run for *H. verticillata* (**I**) and the standard (**VI**) for 34 min at a flow rate of 1 mL/min. at wavelength 290 nm at an ambient temperature of 18 °C. The mobile phase consisted of methanol and water using gradient elution: 20% methanol for 2 min increased to 60% linearly over 4 min and held for 2 min. Methanol was further increased to 80% linearly over 9 min and held for 7 min. The column was washed by running 100% methanol for 8 min then decreased to 20% to equilibrate the column for the next injection.

The fifteen LC-MS spectral results cross-referenced with data from the RP-HPLC analysis using standards (Table 1) (Appendix A, Figure A1 and Figure A2 & Appendix B, Table A1) confirmed the presence of five lignans, deoxydehydropodophyllotoxin, 4′-demethylpodophyllotoxin, (-)-yatein, dehydropodophyllotoxin and podophyllotoxin (Table 1, Figure 3). RP-HPLC analysis with standards alone, confirmed the presence of one further phytochemical, the triterpene oleanolic acid (Table 1, Figure 3) (Appendix A, Figure A2 and Appendix B, Table A1).

### 2.5. Inhibition of CYP1A2 Activity by Key Phytochemicals Present in H. verticillata 

Key phytochemicals were individually assessed for their impact on CYP1A2 activity. Best-fit non-linear plots were fitted to the data points using the 4-parameter logistic (4PL) non-linear regression model (SigmaPlot) and IC_50_ values determined, where possible. Calculated and/or estimated IC_50_ values are summarized in Table 2.

#### 2.5.1. Key Phytochemicals—Mixed Lignans, (-)-Yatein and β-Sitosterol 

The mix of five lignans demonstrated moderate inhibition against the activity of CYP1A2 (61.8 µg/mL). In comparison *H. verticillata* aqueous extract (dried aerial plant material) demonstrated potent inhibition of CYP1A2 activity (1.9 µg/mL). The IC_50_ values determined for (-)-yatein (71.9 µM), demonstrated moderate inhibition, and β-sitosterol (160.1 µM) demonstrated weak inhibition against the activity of CYP1A2. In comparison, the plot and the IC_50_ value for known CYP1A2 inhibitor, furafylline (1.4 µM), demonstrated potent inhibition (Table 2).

#### 2.5.2. Key Phytochemicals—Oleanolic and Ursolic acid, Cadina-4,10(15)-Dien-3-One, Podophyllotoxin, 4′-Demethylpodophyllotoxin and Rosmarinic Acid

It was not possible to determine the IC_50_ values for oleanolic and ursolic acid or cadina-4,10(15)-dien-3-one. However, it was possible to indicate that IC_50_ values for both oleanolic and ursolic acid would be >100 µM and for cadina-4,10(15)-dien-3-one >500 µM, demonstrating weak inhibition against the activity of CYP1A2 (Table 2). Two phytochemicals, podophyllotoxin and 4′-demethylpodophyllotoxin demonstrated no impact on the activity of CYP1A2 (Table 2). It was not possible to plot a graph for rosmarinic acid, due to significant levels of metabolite quenching (>20%) even at lower concentrations (Table 2).

#### 2.5.3. Inhibition of CYP1A2 Activity—Summary

## 3. Discussion

In vitro CYP assays offer an accurate, and relatively inexpensive, first stage assessment tool for gauging the potential for herb-drug interactions. They are useful for initial risk assessment of medicinal plants capable of causing adverse drug reactions (ADRs) when taken concomitantly with pharmaceutical drugs metabolised by the same enzyme [22].

CYPs 1A2, 2D6, and 3A4 are three of the key enzymes involved in drug metabolism, and together, are responsible for the CYP mediated metabolism of approximately 83% of marketed pharmaceuticals. Broken down, the figures are 50% for CYP3A4, 25% for CYP2D6, and 8% for CYP1A2 [23]. CYP3A4 is the enzyme most implicated in drug interactions, while CYP2D6 is important, firstly because of the high percentage of drugs metabolized and secondly, because the enzyme is associated with high levels of polymorphism [8,24]. CYP1A2 is important, as it metabolizes many commonly prescribed drugs including the bronchodilator theophylline, the tricyclic antidepressant imipramine, the beta blocker propranolol and the antipsychotic clozapine [24].

Published results, including those previously reported from our laboratory, point to a general consensus on the levels of inhibition that constitute potent, moderate, and weak inhibition in relation to the *in vitro* inhibition of crude extracts of medicinal plants on human CYP enzymes [15,16,25]: Potent: ≤9.9 µg/mL; Moderate (mild): 10–99.9 µg/mL; Weak: ≥100 µg/mL.

The crude dried aerial aqueous extract of *H. verticillata* demonstrated potent inhibition against the activity of all four CYP enzymes successfully screened, CYPs 1A1, 1A2, 1B1 and 3A4 (Figure 1). Such potent inhibition against recombinant CYP enzymes points to the potential for metabolism-based drug interactions in vivo and requires further investigation for confirmation of clinical relevance. In vitro indications are accepted as a useful initial screen for selecting potential candidates worthy of in vivo evaluations.

Additional research, undertaken to assess the bioactivity of other traditionally prepared extracts, aqueous (fresh) and ethanol (dry and fresh), against the activity of the CYP enzyme most potently inhibited, in this case CYP1A2, demonstrated similar results to those originally obtained with the aqueous extract (dry plant material). The exception was the moderate level of inhibition seen for the aqueous extract of the fresh plant material (27.8 µg/mL). This is most likely due to the lower concentration of plant material on a weight by volume basis in the aqueous extract prepared with fresh plant material. Fresh plant material has a higher water content, and as a result approximately 3–4 g of fresh plant material is required to provide the equivalent weight of 1 g of dried material [26].

CYP1A2 is an important enzyme, because it is responsible for metabolizing a number of commonly prescribed drugs, several of which are used in psychiatric medicine—drugs such as fluvoxamine, amitriptyline, clomipramine and clozapine. Several of these drugs have narrow therapeutic ranges, which consequently increases the risk of serious ADRs when interactions occur [23]. The potent inhibition of this important drug metabolizing enzyme by the aqueous (dried aerial material) and ethanol (fresh and dried aerial material) extracts of *H. verticillata* indicate the potential for pharmacokinetic metabolism-based drug interactions and warrants further in vivo investigations [8,27].

In addition, certain CYP enzymes, particularly the CYP1 family, play a role as potential cancer promoting agents. Potent inhibitors of these enzymes, *in vitro,* have chemopreventive potential *in vivo,* and warrant further investigation [28,29,30,31]. CYP1A1 is particularly implicated in lung, colorectal, breast, and prostate cancers, CYP1B1 in hormone dependent cancers of the prostate, breast, and endometrium, and CYP1A2 in colorectal, lung, and breast cancers [29,31,32]. Selectivity is an important criterion in short-listing plants for further assessment. It is notable that natural products successfully patented for their potential therapeutic value tend to demonstrate high selectivity in their inhibition of specific CYP activities [33].

Such likely chemopreventive properties are often corroborated by the antioxidant activities displayed by extracts. The four tested extracts demonstrated strong DPPH scavenging activity with IC_50_ values ranging from 1.6 µg/mL to 7.4 µg/mL (Figure 2). This strong antioxidant activity, together with the plant’s potent *in vitro* inhibition of CYPs 1A1 and 1B1 (Figure 1), known to be potent activators of carcinogens, points to potential chemopreventive properties and warrant further in vivo investigation [8,28,29,30,31].

Similar antioxidant research on a number of other plants from the Lamiaceae family, reported IC_50_ values ranging from 3–44 µg/mL. Plant material was prepared using aerial dried samples in ethanol and results were compared to the reference antioxidant, Trolox (a vitamin E analog) (1.99 µg/mL), using the DPPH assay. One of the plants reported, *Rosmarinus officinalis*, often used as a positive control for antioxidant research, had an IC_50_ value of 5.0 µg/mL [34].

The antioxidant activity of *H. verticillata* was previously reported by Williams et al. [35] for extracts prepared from dried aerial plant material in ethanol and using the DPPH assay. On this occasion, the researchers reported antioxidant activity as percentage activity relative to control with reported results ranging from 0% to 96%. Of the plants screened, seven were from the Lamiaceae family and their reported antioxidant activities ranged from 17% to 55% with *H. verticillata* and *Rosmarinus officinalis* both reporting antioxidant activities of 55%.

Further investigations were undertaken to characterise the phytochemicals in the crude aqueous extract of the dried aerial plant material of *H. verticillata,* to identify the key phytochemical(s) responsible for the inhibition of CYP1A2, and to allow further assessment of the plant preparation’s potential to impact the activities of CYP enzymes in vivo.

The combined use of RP-HPLC and LC-MS confirmed the presence of five phytochemicals. These were the five lignans, podophyllotoxin, 4′-demethylpodophyllotoxin, (-)-yatein, dehydropodophyllotoxin and deoxydehydropodophyllotoxin, the latter two being part of the five-lignan mixture (Table 1, Figure 3).

The presence of the triterpene, oleanolic acid was confirmed by RP-HPLC alone (Table 1, Figure 3). The two triterpenes, oleanolic acid and ursolic acid exist as regioisomers, with the only difference between them being the position of one methyl group. Whilst ursolic acid was not directly analysed to confirm its presence, based on the reports that both triterpenes exist together in plants simultaneously, it is highly likely that ursolic acid is also present [36,37].

Five individual phytochemicals, together with the mixture of five lignans were tested for their impact on the activity of CYP1A2. The lignan mix and one individual phytochemical, (-)-yatein, demonstrated moderate/weak inhibition, with IC_50_ values of 61.8 µg/mL and 71.9 µM, respectively. The two triterpenes, oleanolic acid and ursolic acid, demonstrated weak inhibition, with IC_50_ values >100 µM. The remaining two individual phytochemicals, the lignans podophyllotoxin and 4-demethylpodophyllotoxin demonstrated no inhibition of CYP1A2 activity (Table 2).

Two further phytochemicals, cadina-4,10(15)-dien-3-one and ß–sitosterol were found to be weak inhibitors of CYP1A2 (Table 2). Their presence was ruled out through RP-HPLC screening with standards, however if they had been found to be present, their weak inhibition would indicate that they would not have contributed to the potent inhibition seen with the aqueous extract of *H. verticillata* (dried aerial plant).

By way of comparison, an assay with a known CYP1A2 inhibitor, furafylline, was tested against the activity of CYP1A2, and an IC_50_ value of 1.4 ± 0.15 μM generated, which was consistent with values previously obtained and published by our laboratory (Table 2) [15].

Table 3 summarises the impact of a number of the key phytochemicals, identified in the aqueous extract of *H. verticillata* (dried aerial material), on CYP enzyme activity where this has been independently reported in the literature. Only two of the phytochemicals, oleanolic and ursolic acid, appear to have been previously screened against the activity of CYP1A2 [38]. In both cases, the reported inhibition was weak, >100 μM, in line with the results now being reported in our laboratory.

Podophyllotoxin and (-)-yatein are reported as potent inhibitors of CYP3A4 [39,40] and (-)-yatein is reported as a moderate/weak inhibitor against the activity of CYP2D6 [39] (Table 3). Our results, reporting no inhibition by podophyllotoxin and moderate/weak inhibition by (-)-yatein against the activity of CYP1A2 for the first time, further strengthens the earlier reports of selectively potent inhibition of CYP3A4 by these two phytochemicals (Table 2).

Having confirmed the identity of seven phytochemicals in the aqueous extract of *H. verticillata* (dried aerial material), and screened them for their impact on the activity of CYP1A2, it appears that the bioactivity displayed by the crude aqueous extract of the plant (dried aerial material) can be attributed in part to yatein and two of the phytochemicals identified from the mixture of lignans, which displayed moderate/weak and moderate inhibition, respectively, of the same enzyme. The potential also remains for other unidentified contributors, along with synergistic activities. Whilst research on many medicinal plants has been able to identify and link one key phytochemical, or class of phytochemicals, to the bioactivity of a plant, an alternative explanation is that of a synergistic effect. This is where a number of phytochemicals act together synergistically to contribute to the observed bioactivity, a fact previously noted and attributed to a number of the documented pharmacological effects and traditional uses of *H. verticillata* [41]. Initial attempts made to quantify synergy using isobolograms proved inconclusive at this stage, however the investigations would benefit with an extension using a wider cocktail of phytochemicals. A growing body of research into synergistic effects is reported in the literature [42,43,44]. Research, and the understanding of synergistic effects, is not limited solely to natural products, but is also gaining recognition in the emerging fields of network pharmacology and compound synergy [45,46,47,48].

In conclusion, this research presents the results of an in-depth assessment of the medicinal plant *H. verticillata*, its impact on a number of key CYP450 enzymes, antioxidant properties, and its characterisation and further impact on CYP1A2. The results have confirmed the identity of seven phytochemicals in the aqueous extract prepared from the dried aerial plant material, five lignans and two triterpenes.

Four traditional preparations of the aerial plant material demonstrated strong antioxidant activity, which, together with the plant extracts potent inhibition of known carcinogen activators, CYPs 1A1 and 1B1, and previously reported anti-inflammatory and anticancer properties [19] warrants further research into the potential chemopreventive properties of this interesting plant, bearing in mind the knowledge of its inhibitory impact on drug metabolizing CYP enzymes.

Screening phytochemicals against the activity of CYP1A2 identified yatein as a moderate-weak contributor to the plant’s potent bioactivity, with potential for synergistic activity among other constituents. Further analysis on the potential impact of *H. verticillata* on key drug metabolizing enzymes in vivo appears warranted given the extensive use of this plant, both in Jamaica and across the Caribbean region.

## 4. Material and Methods

### 4.1. Reagents

CYP substrates and metabolites were purchased from BD Gentest Corporation (Woburn, MA, USA). All other chemicals were purchased from Sigma Aldrich (St. Louis, MO, USA), unless otherwise stated. β-Sitosterol, cadina-4,10(15)-dien-3-one and a mix of five lignans comprised of dehydropodophyllotoxin, deoxydehydropodophyllotoxin, 4′-demethyldesoxypodophyllotoxin, 5′-methoxydehydropodophyllotoxin, and dehydro-b-peltatin methyl ether were supplied by the Department of Chemistry, University of the West Indies, Mona Campus, Jamaica. 4′-Demethyl-podophyllotoxin and (-)-yatein were purchased from BOC Sciences (New York, NY, USA), oleanolic acid, ursolic acid, and rosmarinic acid were purchased from Cayman Chemical (Ann Arbor, MI, USA) and podophyllotoxin was purchased from Sigma Aldrich. LC-MS grade high purity water and LC-MS grade high purity methanol were purchased from Sigma Aldrich (Gillingham, UK). 2,2-diphenyl-1-picrylhydrazyl (DPPH) was purchased from Sigma Aldrich (St. Louis, MO, USA).

### 4.2. Co-Expressed Human CYP Enzymes 

*Escherichia coli* membranes expressing human CYP1A1, CYP1A2, CYP1B1, CYP3A4, and CYP2D6, co-expressed with CYP reductase, were purchased from Cypex Ltd. (Dundee, UK).

### 4.3. Preparation of Crude Plant Extracts 

Samples of the plant material were collected, and voucher specimens prepared (voucher number 35473) and deposited with the Herbarium at the University of the West Indies, Mona, Jamaica, where the identity of the plant was confirmed by Mr. Patrick Lewis, botanist and Herbarium Curator.

#### 4.3.1. Aqueous Extracts—Dried Plant (Leaf and Stem)

The following method of preparation was previously developed and refined in our laboratory [10,16]. Collected leaf and stem plant material was bench-dried in our laboratory and then finely crushed using a coffee grinder. The ground plant material was prepared as an infusion following traditional Jamaican practices [17,19] using 100 mL of boiled deionized water per 1 g of dried plant material and infused for fifteen to twenty minutes. The resulting liquor was suction filtered through type 1 Whatman filter paper and centrifuged (MSE Micro Centaur, Sanyo, Osaka, Japan) at 13,000 g for five minutes to remove suspended solids. The samples were frozen at −20 °C in round bottom flasks and lyophilised using a freeze drier (Labconco, Kansas City, MO, USA). The resulting solids were placed in vials and kept at −20 °C until required, and not subjected to more than two freeze-thaw cycles.

#### 4.3.2. Aqueous Extracts—Fresh Plant (Leaf and Stem) 

The fresh aerial parts (leaf and stem) of *H. verticillata* were roughly cut and prepared as infusions without drying. All other details of the methodology employed were the same as those detailed above.

#### 4.3.3. Ethanol Extracts—Dried and Fresh Plant (Leaf and Stem)

Ethanol extracts (tinctures) were prepared from fresh and dried plant material (leaf and stem) using documented traditional methods [17,19]. Dried plant material was bench-dried in the laboratory, finely chopped in a coffee grinder and placed into a sealable, darkened glass container. The tincture was prepared by maceration at room temperature using 10 mL of 75% ethanol (Sigma Aldrich, USA) per gram of dried plant material. The sealed container was shaken and turned daily for a period of two weeks at room temperature. The resulting liquor was suction-filtered through type 1 Whatman filter paper and stored in a sealed darkened glass container. The resulting extract was a 1:10 (*w*/*v*) (75% EtOH) standard tincture, in which 10 mL of the final preparation is equivalent to 1 g of the dried plant from which the preparation was made. Fresh plant material (leaf and stem) was roughly cut and placed into a darkened sealable glass container. The tincture was prepared as above. The resulting extract was a 1:10 (*w*/*v*) (75% EtOH) specific tincture (fresh plant material).

### 4.4. Standardization of Dried Plant Aqueous Extract (Leaf and Stem) by Reversed Phase HPLC (RP-HPLC) and LC-MS

Analysis of *H. verticillata* was undertaken to characterise the aqueous extract prepared from the bench-dried aerial parts (leaf and stem) of the plant. The following chromatographic methods were employed.

#### 4.4.1. High Performance Liquid Chromatography (HPLC)

RP-HPLC analysis was carried out on a Series 200 instrument (Perkin Elmer, Waltham, MA, USA) equipped with a quaternary pump, thermostatted autosampler, column oven, and diode array detector. The column used was an RP-18 reversed phase ChromoSep HPLC SS (250 × 4.6 mm, 5 μm) (Varian, Palo Alto, CA, USA) including s holder with an Intersil ODS-2 ChromoSep guard column. Appropriate methodologies were developed based on previous analysis of the plant in the Department of Chemistry, UWI, [20] and on published methodologies for key phytochemicals previously identified in the aerial parts of *H. verticillata* [36,53].

#### 4.4.2. Liquid Chromatography—Mass Spectrometry (LC-MS)

The LC–MS system used comprised a 1260 Infinity LC system (Agilent Technologies, Craven Arms, UK) coupled to a 6430 triple quadrupole mass spectrometer (Agilent Technologies). The LC system consisted of a 1290 infinity thermostatted autosampler, degasser, binary pumps, and column heater. An electrospray ionisation (ESI) source was used for sample analysis. The sample was injected through a Zorbax Eclipse plus C18 (2.1 × 100 mm, 1.8 μm) column. The column was heated to 50 °C for good reproducibility. The analytical column was connected in tandem with a 0.2 μm inline filter to prevent it from blocking. The mobile phase consisted of solvent A, LC-MS grade high purity water and solvent B, LC-MS grade high purity methanol. A gradient elution was used for analysis whereby 20% methanol was run for 2 min and then increased to 60% methanol linearly over 4 min and held for 2 min. Methanol was further increased to 80% linearly over 9 min and held for 7 min. The column was washed by running 100% methanol for 8 min then decreased to 20% methanol for 2 min to equilibrate the column for the next injection.

The flow rate through the column was set at 0.4 mL/min. The total run time was thirty-four minutes. A sample (20 µL) was injected on to the LC-MS system. Auto needle wash was set up to remove any carryover effects in the LC analysis. The bypass configurations were set up for the mixer and damper with Agilent HPLC 1260 binary pump. This was to convert the pump to low delay volume mode and better reproducibility. Both positive and negative ESI were utilised in Full Scan mode. Positive ESI was selected for further analysis because it gave more prominent ion peaks in the full scan mode. The mass spectrometer was operated in the positive ionization polarity made at a spray capillary voltage of 3000 V. Sheath gas temperature and flow were 350 °C &12 L/min resp., nozzle voltage was 450 V, drying gas temperature and flow 300 °C & 8 L/min resp., nebulizer gas pressure was 25 psi.

High resolution LC-MS was conducted using a Waters LCT Time of Flight Mass Spectrometer (Waters Micromass Ltd., Wilmslow, UK), with a Waters Alliance 2690 HPLC and Mass Lynx V4.1 software using similar HPLC conditions to those described above.

### 4.5. In Vitro Inhibition of CYP Activity

The following methodology was developed by Crespi et al. [54] and adapted by Shields et al. [16] and Murray et al. [15] to screen natural products for cytochrome P450 inhibition potential with a particular emphasis on the P450 enzymes, CYPs 1A1, 1A2, 1B1, 3A4 and 2D6. Plant extracts and various phytochemicals were evaluated for their ability to inhibit the catalytic activity of human cytochrome P450 enzymes by means of high throughput fluorometric inhibition assays conducted in 96 well microtitre plates as described by Crespi et al., BD Gentest [54] and Murray et al. [15].

7-Ethoxy-3-cyanocoumarin (CEC) was used as a substrate for detecting the activities of CYPs 1A1, and 1A2, 7-ethoxyresorufin (ERes) for CYP1B1, 7-benzyloxy-4-trifluoromethylcoumarin (BFC) for CYP3A4, and 3-[2-(*N*,*N*-diethyl-*N*-methylammonium)-ethyl]-7-methoxy-4-methylcoumarin (AMMC) for CYP2D6. The reactions were monitored fluorometrically at 37 °C, using an Eclipse Fluorescence Spectrophotometer (Varian Cary, Palo Alto, CA, USA).

### 4.6. Controls Assessment

#### 4.6.1. Positive Control Inhibition for CYP450 Assays (CYPs 1A1, 1A2, 1B1, 2D6, 3A4)

Positive control experiments were conducted with known inhibitors with varying concentrations of *α*-naphthoflavone for CYPs 1A1 and 1B1, furafylline for CYP1A2, quinidine for CYP2D6, and ketoconazole for CYP3A4.

#### 4.6.2. Solvent Impact on CYP1A2 Activity

In assaying the inhibitory effect of materials on CYP activity, the choice of solvent systems is important. The activity of CYP enzymes is known to be significantly inhibited or induced by certain non-aqueous solvents at higher concentrations [55,56]. Dimethylformamide (DMF) has been identified as one of the least inhibitory solvents towards CYP1A2 [55]. Control assays run with DMF confirmed that no inhibition against CYP1A2 occurred at concentrations levels below 3%, demonstrating the least impact with no significant levels of inhibition or potentiation of fluorescence across the range of concentrations tested.

#### 4.6.3. Intrinsic Fluorescence

Each of the various crude extracts, shortlisted phytochemicals and solvents (DMSO, DMF and ethanol) were assessed for intrinsic or natural fluorescence to ensure no interference with known metabolite fluorescence at each of the relevant excitation and emission wavelengths.

#### 4.6.4. Metabolite Fluorescence Quenching

Each of the various crude extracts, shortlisted phytochemicals and solvents (DMSO, DMF and ethanol) were investigated for their potential levels of quenching against the fluorescence of each known metabolite. Concentrations that resulted in quenching were avoided in subsequent screenings.

### 4.7. Antioxidant Assay 

The DPPH radical scavenging capacity of extracts of *H. verticillata* were evaluated by the method described by Williams et al. [35]. The electron-donating ability of each extract, at varying concentrations, was measured colourimetrically through the bleaching of purple-coloured MeOH solution of DPPH. The known antioxidant, ascorbic acid, was used as a positive control.

Each test extract, as well as the ascorbic acid control, was made up in methanol to a starting concentration of 50 µg/mL and serially diluted in ninety-six well microtitre plates. 100 µL of various concentrations of the extracts, and the ascorbic acid control, in methanol, were added to 100 µL of a 0.02% (*w*/*v*) stock solution of the free radical 2,2-diphenyl-1-picrylhydrazyl (DPPH) in methanol. After a thirty-minute incubation period at room temperature, the absorbance was read against a blank, comprising methanol, at 517 nm. All tests were carried out in duplicate for at least two independent experiments.

### 4.8. Data Analysis

#### 4.8.1. Measuring Cytochrome P450 Inhibition

IC_50_ values (concentration at which 50% of activity remains) were determined by fitting data in SigmaPlot (version 10.0), using the four parameter logistic (4PL) non- linear regression model. The data listed represent average values from a minimum of two experiments run in triplicate:

% Enzyme activity = [Min + (Max − Min)/(1+10^((LogIC^_50_^− x) × p)^)] where Min and Max are the minimal and maximal observed effects, respectively; x is the concentration of test agent; EC_50_ is the concentration yielding half-maximal response (i.e., IC_50_); and p is the slope parameter.

#### 4.8.2. Antioxidant Assay

The level of DPPH inhibition was calculated according to the formula:

Inhibition (%) = [(**A**_blank_ – **A**_sample_)/**A**_blank_ ] × 100 where **A**_blank_ is the absorbance of the control reaction (containing all reagents except the test compound) and **A**_sample_ is the absorbance of the test compound.

IC_50_ values were determined by fitting the data in SigmaPlot (version 10.0, San Jose, CA, USA) using the four parameter logistic (4PL) non-linear regression model. An extract concentration providing 50% inhibition (IC_50_) was calculated from the graph plotted as inhibition percentage against extract concentration.

## Figures and Tables

**Table 1:** LC-MS * and RP-HPLC analysis and summary.

Phytochemicals Identified in *H. verticillata* by LC-MS	Empirical Formula	Mono-isotopic Mass (Da) ^1^	Confirmation by RP-HPLC Standard
Cadina-10(15)-en-3-one	C_15_H_22_O	218.167	
Aromadendr-1(10)-en-9-one	C_15_H_22_O	218.167	
7,11,15-Trimethyl-3-methylenehexadecane -1,2-diol	C_20_H_40_O_2_	312.303	
Rosmarinic acid	C_18_H_16_O_8_	360.315	
**Deoxydehydropodophyllotoxin ^2^**	**C_22_H_18_O_7_**	**394.105**	**(+)**
Dehydrodesoxypodophyllotoxin	C_22_H_18_O_7_	394.105	
ß-Apopicropodophyllin	C_22_H_20_O_7_	396.121	
Hyptinin	C_22_H_20_O_7_	396.121	
Isodeoxypodophyllotoxin	C_22_H_22_O_7_	398.136	
Deoxypicropodophyllin	C_22_H_22_O_7_	398.136	
**4-Demethylpodophyllotoxin **	**C_21_H_20_O_8_**	**400.116**	**(+)**
**(-)-Yatein **	**C_21_H_20_O_8_**	**400.152**	**(+)**
**Dehydropodophyllotoxin ^2^**	**C_22_H_18_O_8_**	**410.100**	**(+)**
**Podophyllotoxin **	**C_22_H_22_O_8_**	**414.131**	**(+)**
ß-Peltatin	C_22_H_22_O_8_	414.131	
Oleanolic acid ^3^	C_30_H_48_O_3_	456.360	(+)

^1^ ChemSpider—Royal Society of Chemistry; ^2^ Lignans in the five lignan mix supplied by UWI Dept. of Chemistry; (+) tested and confirmed to be present using HPLC analysis with the relevant standard; **Bold**—five phytochemicals identified through both LC-MS and RP-HPLC analysis; ^3^ Phytochemical identified through RP-HPLC alone; * Exact mass measurements using a High Resolution instrument (Waters LCT ToF MS, with a Waters Alliance 2690 HPLC and Mass Lynx V4.1 software).

**Table 2:** IC_50_ values of the aqueous extract of *H. verticillata* and key phytochemicals and their impact on metabolism mediated by CYP1A2.

**Test Phytochemical**	**Concentration Range Tested [µg/mL]**	**Solvent (Highest % *v*/*v*)**	**IC_50_ (µg/mL)**
*H. verticillata* aqueous extract (dried aerial material)	0.25–60	H_2_O	1.9 ± 0.5
Lignan Mix	0.08–180	DMF (2.0)	61.8 ± 0.7
-Dehydropodophyllotoxin -Deoxydehydropodophyllotoxin -4′-Demethyldesoxypodophyllotoxin -5′-Methoxydehydropodophyllotoxin -Dehydro-β-peltatin methyl ether			
**Test Phytochemical**	**Concentration Range Tested [µM]**	**Solvent (Highest % *v*/*v*)**	**IC_50_ (μM)**
Podophyllotoxin	2.9–369	DMF (0.7)	No inhibition
4′-Demethylpodophyllotoxin	0.28–611	DMF (0.7)	No inhibition
(-)-Yatein	0.18–400	DMF (1.2)	71.9 ± 1.8
Cadina-4,10(15)-dien-3-one	0.28–623	H_2_O	>500
Oleanolic acid	0.9–219	DMF (1.3)	>100
Ursolic acid	0.05–117	DMF (1.8)	>100
β-Sitosterol	5.1–410	DMF (2.0)	160 ± 3.4
Furafylline—reference inhibitor	0.04–96		1.4 ± 0.15

**Table 3:** Review of published CYP inhibition research for key phytochemicals.

Phytochemicals	Reported CYP Inhibition	IC_50_ Value (μM)	Reference
Podophyllotoxin	CYP3A4	Potent inhibitor (IC_50_ not stated)	[40]
Dehydropodophyllotoxin	None	-	
Deoxydehydropodophyllotoxin	None	-	
4′-Demethyldesoxypodophyllotoxin	None	-	
4′-Demethylpodophyllotoxin	None	-	
5′-Methoxydehydropodophyllotoxin	None	-	
Dehydro-β-peltatin methyl ether	None	-	
(-)-yatein	CYP3A4 CYP2D6	1.095.7	[39] [39]
Oleanolic acid	CYP1A2 CYP2C8/C9/C19 CYP3A4 CYP2D6	143.5 >500 78.9 >500	[38] [38] [38] [38]
Ursolic acid	CYP1A2 CYP2C8/C9/C19 CYP3A4 CYP2D6	352.4 >500 >500 438.9	[38] [38] [38] [38]
Rosmarinic acid	CYP2C9 CYP2D6 CYP3A4 CYP3A4	318.9 >500 241.2 No impact	[49] [49] [49] [50]
ß-Sitosterol	CYP2D6 CYP3A4 CYP3A4/3A5 CYP2C19	>100 >100 No impact No impact	[51] [51] [52] [52]

## Appendix B

**Table A1:** LC-MS and HPLC analysis and summary.

Phytochemicals Identified in *H. verticillata* (Published and Unpublished)	RP-HPLC Standard	Empirical Formula	*m/z*—Calculated Value ^1^ (Difference Between *m/z* Value and Mono-Isotopic Mass Where the Difference is ≤1.0)					
			[M + H] ^+^	[M + CH_3_] ^+^	[M + H_2_O] ^+^	[M + Na] ^+^	[M + CH_3_OH] ^+^	[M + K] ^+^
Cadina-4,10(15)-dien-3-one	(−)	C_15_H_22_O						
Cadina-10(15)-en-3-one		C_15_H_22_O						
Aromadendr-1(10)-en-9-one		C_15_H_22_O						
7,11,15-Trimethyl-3-methylenehexadecane -1,2-diol		C_20_H_40_O_2_			330.3 (0.9)			
Rosmarinic acid		C_18_H_16_O_8_					392.3 (0.97)	
Deoxydehydropodophyllotoxin ^2^	(+)	C_22_H_18_O_7_		409.1 (0.1)				433.1 (0.7)
Dehydrodesoxypodophyllotoxin		C_22_H_18_O_7_		409.1 (0.1)				433.1 (0.7)
ß-Apopicropodophyllin		C_22_H_20_O_7_				419.0 (0.4)		
Hyptinin		C_22_H_20_O_7_				419.0 (0.4)		
Isodeoxypodophyllotoxin		C_22_H_22_O_7_						437.1 (0.2)
Deoxypicropodophyllin		C_22_H_22_O_7_						437.1 (0.2)
4-Demethylpodophyllotoxin	(+)	C_21_H_20_O_8_			418.1 (0.3)		432.1 (0.3)	
(-)-Yatein	(+)	C_21_H_20_O_8_			418.2 (0.2)		432.2 (0.2)	
Dehydropodophyllotoxin ^2^	(+)	C_22_H_18_O_8_				433.0 (0.6)		
Podophyllotoxin	(+)	C_22_H_22_O_8_			432.1 (0.3)	437.0 (0.3)		
ß-Peltatin		C_22_H_22_O_8_			432.1 (0.3)	437.0 (0.3)		
ß -Sitosterol ^3^	(−)	C_29_H_50_O			432.4 (0.0)	437.3 (0.0)		
Oleanolic acid ^4^	(+)	C_30_H_48_O_3_						

^1^ Mass difference (predicted to measured) in brackets using a High Resolution instrument (Waters LCT ToF MS, with a Waters Alliance 2690 HPLC and Mass Lynx V4.1 software); ^2^ Lignans in the five lignan mix supplied by UWI Dept. of Chemistry; ^3^ Metabolite identified in *H. verticillata*, but not published at the time of review; ^4^ Phytochemicals identified through RP-HPLC alone (not LC-MS); (+)(-) tested and confirmed to be present or absent using HPLC analysis with the relevant standard
